# Immune profiling by bulk RNA-seq and multiplex immunofluorescence imaging across multiple cancer types

**DOI:** 10.3389/fonc.2026.1876853

**Published:** 2026-08-06

**Authors:** Catia Gaspar, Benson Wu, Simone C. Stone, Valentin Sotov, Ben Wang, Collins Wangulu, Hal Berman, Aileigh Kay, Prabhjit Basra, Emily Van de Laar, Scott Bratman, Stephanie Lheureux, Jennifer Knox, Raymond Kim, Hansen He, Geoffrey Liu, Philippe Bedard, Anna Spreafico, Ming Tsao, Trevor J. Pugh, Aaron Hansen, Gonzalo Sapisochin, Enrique Sanz Garcia, Lillian L. Siu, Jeffrey P. Bruce

**Affiliations:** 1 Division of Medical Oncology and Hematology, Princess Margaret Cancer Centre, University Health Network, University of Toronto, Toronto, ON, Canada; 2 University Health Network , University of Toronto, Toronto, ON, Canada; 3 Princess Margaret Cancer Centre, University Health Network, University of Toronto, Toronto, ON, Canada; 4 Princess Margaret Cancer Consortium, Marathon of Hope Cancer Centres Network, Toronto, ON, Canada

**Keywords:** immunotherapy, multiplexed IHC, pan-cancer analysis, RNA sequencing, tumor micro environment (TME)

## Abstract

**Background:**

Immune cell populations within the tumor microenvironment (TME) critically determine cancer prognosis and therapeutic response. Although multiplexed immunohistochemistry (mIHC) provides spatial immune profiling and bulk RNA sequencing methods offer comprehensive cellular characterization, comparative studies between these complementary methodologies remain limited across diverse tumor types.

**Methods:**

Patient samples from various tumor types, obtained from either primary or metastatic sites, were collected. For each patient, two samples were analyzed, one for mIHC and one for RNA-seq (xCell2). Both methodologies were intended to quantify immune cell populations including CD4 and CD8 T cells, regulatory T cells (Tregs), B cells and macrophages within the TME. Samples were stratified by origin according to biopsy procedure and tissue block characteristics. The primary objective was to assess concordance between methodologies for immune cell density estimation, while exploratory analysis evaluated liver-specific TME differences and immunotherapy (IO) exposure in head and neck squamous cell carcinoma (HNSCC) patients. Method correlations were analyzed using Spearman’s rank correlation coefficient.

**Results:**

Among 298 patient specimens spanning 20 tumor types, overall correlation between mIHC and xCell2 deconvolution was modest (*ρ* 0.35 – 0.50), due to sample heterogeneity, with CD8 T cells demonstrating the strongest correlation (*ρ* = 0.50). Tumor-specific analysis revealed enhanced correlations in HNSCC (*ρ* = 0.80) and renal clear cell carcinoma (*ρ* = 0.85) for CD8 T cells, while primary tumor samples showed improved CD8 T-cell correlation compared to metastatic sites (*ρ* = 0.60). Macrophage density was significantly elevated in primary hepatocellular carcinoma versus liver metastases (p = 0.0336). Within the HNSCC subgroup, IO-exposed samples exhibited significantly reduced CD4 T-cell (p = 0.039) and B-cell (p = 0.022) infiltration compared to treatment naïve specimens.

**Conclusions:**

Our work highlights the value of integrating spatial (mIHC) and transcriptomic (xCell2) approaches to optimize immune profiling accuracy in complex tumor tissues. CD8 T-cell populations showed the strongest methodological concordance, establishing their priority as a robust candidate biomarker for immune activation to be used in further clinical research.

## 1 Background

Recent advancements in cancer research have underscored the utility of characterizing immune cell subtypes within the tumor microenvironment (TME) to identify potential prognostic and predictive biomarkers for anticancer therapies, particularly immunotherapy (IO). Standard pathology reports currently do not provide information about immune infiltrates, leaving this potentially valuable data unexplored. Recent progress in immuno-oncology has underscored the importance of the TME and the intricate interactions between cancer cells and the immune system. The TME is a complex ecosystem that provides a supportive framework for tumor growth and progression, comprising immune cells, stromal cells, blood vessels, and extracellular matrix (1). Immune components constitute a critical and dynamic element of the TME, encompassing various cell types such as T cells, B cells, natural killer cells, macrophages, neutrophils, and dendritic cells. These immune cells within the TME exhibit a dual nature, capable of exerting both pro-tumorigenic and anti-tumorigenic effects, thus playing a role in either promoting or suppressing tumor growth (2).

The immunological landscape of tumors has emerged as a critical determinant in both prognostic assessment of disease outcomes and prediction of treatment efficacy across diverse malignancies, gaining recognition as a key hallmark of cancer (3). The prognostic utility of immune cells within the TME has been extensively studied in several cancer types, particularly in lung cancer, melanoma, and head and neck squamous cell carcinoma, where immunotherapies have revolutionized cancer care (4, 5). The influence of the TME on treatment response has been investigated across various cancer types, including those from endometrial and ovarian, genitourinary tract, breast, brain, and gastrointestinal tract carcinomas ^(^6–8^),^. Notably, in colorectal cancer, immunoprofiling data have demonstrated superior predictive value for patient survival compared to traditional histopathological staging methods (9). These findings underscore the potential importance of TME characterization in refining prognostic assessments and treatment strategies across multiple cancer types, suggesting that immune profiling may complement or even surpass conventional staging techniques in certain contexts.

Immune resistance often results from TME changes due to acquired mechanisms that alter the infiltration of immune cells and decrease drug penetration capacity, leading to immunoediting of cancer cells. The impact of TME on treatment response can also be seen with therapeutic drug classes like tyrosine kinase inhibitors, which can modulate immune responses and enhance antitumor effects (10). Current therapeutic strategies targeting the TME such as vaccines (11), oncolytic viruses (12) and even radiation (13), have the ability to modulate various immune cell components, including dendritic cells, macrophages, B cells, and T cells (14, 15), with the possibility of converting immunologically “cold” tumors into “hot” tumors. Despite these advancements, no specific immune cell component of the TME has yet been established as a clinically validated biomarker for patient selection in these treatment approaches.

Immune cell subtype estimation has traditionally relied on quantitative immunohistochemistry (IHC) using single antibody markers for each immune cell sub-population. However, the advent of multiplexed IHC (mIHC) has enabled the simultaneous detection of multiple markers on a single tissue section, allowing for a more comprehensive evaluation of TME composition, immune contexture, and cell-cell interactions. Concurrently, in silico tools developed for analyzing bulk RNA-seq data are being employed to characterize the immune compartment in the TME, offering more comprehensive information about immune cell subtypes (16). While the integration of these innovative technologies presents implementation challenges, ongoing research and technological advancements are progressively facilitating their translation into research and ultimately, routine clinical practice.

While correlation between mIHC and RNA-seq analysis for individual marker genes is well-documented in the literature (17), there is limited knowledge regarding their correlation when analyzing TME cell composition. Some research groups have investigated TME estimation and comparison using different methodologies but focusing primarily only on specific cancer types and with relatively small sample sizes (18). Consequently, the overall conclusions drawn from these studies have been inconsistent.

This study aims to analyze the concordance between mIHC and bulk RNA-seq, using paired patient samples from diverse tumor types. The investigation will focus on the technical aspects of both methods, addressing the challenges posed by sample heterogeneity. Additionally, selected clinical correlates will be provided, emphasizing the advantages of each method. The study will explore future strategies that may be employed to effectively select patients for therapies targeting the TME. By comparing these two methodologies across various cancer types, this research seeks to contribute to the development of more robust and reliable approaches for characterizing the TME and potentially improving patient selection for anticancer therapies.

## 2 Methods

In this study, two techniques were employed to analyze the immune component of the TME in patient samples from various tumor types: mIHC and RNA sequencing (RNA-seq). The samples were obtained from patients who provided informed consent to participate in one of the Marathon of Hope Cancer Centres Network (MOHCCN) research projects at Princess Margaret Cancer Centre. The primary objective was to compare the concordance between these two methods in estimating immune cell density, while addressing potential confounding factors that could influence this direct comparison.

### 2.1 Marathon of hope cancer centres network

MOHCCN is a collaborative initiative designed to unite cancer centers and researchers across Canada with the goal of advancing research and precision oncology. A key objective of this network is to develop a comprehensive genomic-transcriptomic and clinical database to support data sharing and integration across approximately 140 projects. The Princess Margaret Cancer Consortium serves as one of the primary contributors to this network, currently leading 43 active projects.

For this study, samples and data were sourced from multiple cohorts encompassing various tumor types. Detailed descriptions of each cohort are provided in **Supplementary Table 1**.

This approach facilitates a robust analysis by leveraging diverse datasets within the framework of the MOHCCN.

### 2.2 Samples retrieval and concordance between samples

Tumor samples were obtained from either primary tumor or metastatic site biopsies; all were available as paraffin-embedded (FFPE) specimens (for RNA-seq and for mIHC) and some were available also as fresh frozen specimens (for RNA-seq in some cases). For each patient, two samples were analyzed, one for mIHC and one for RNA-seq. These samples could originate from the same procedure and tissue block, or they might be derived from different procedures, specimens, or time points. To account for the potential variability in sample origin and timing, each patient/case was classified into a ‘Tier’ based on the degree of concordance between the materials used in each assay. This classification system allows for a more nuanced analysis of the data, taking into consideration the potential impact of sample heterogeneity on the results.

The classification system for sample concordance is structured as follows:

- Tier 1: Samples obtained from the same procedure and the same tissue block, representing the highest degree of concordance between the two samples (n=88).
- Tier 2: Samples originating from the same procedure but different tissue blocks. For instance, both samples might be derived from a surgical resection of a primary tumor, but different blocks were used to obtain material for RNA-seq and for mIHC (n=177).
- Tier 3: Samples derived from different procedures, potentially different time points, and different tumor sites. For example, sample 1 (for bulk RNA-seq analysis) might be obtained from an initial diagnostic biopsy of the primary tumor, while sample 2 (for mIHC analysis) might be obtained from a subsequent biopsy of a metastatic site (n=16).

This study aims to analyze the concordance in TME immune cell composition estimates between two samples for each patient. The analysis is stratified by tier, tumor type and biopsy site (primary vs. metastatic). In addition to the primary analysis, exploratory clinical investigations were conducted. These included an examination of the specific liver microenvironment, aiming to elucidate potential unique characteristics of this tissue context. Furthermore, an investigation was performed to understand the possible effects of IO exposure in head and neck squamous cell carcinoma (HNSCC), derived mainly from oral cavity (OCSC) primary tumors.

Validation of the divergent macrophage infiltration levels identified between primary HCC and secondary liver deposits was performed by analyzing two public transcriptomic datasets: TCGA-LIHC (representing primary HCC) and MET500 (comprising liver metastases). Transcriptome and phenotype data were downloaded from the UCSC Xena Browser (https://xenabrowser.net/datapages/?cohort=GDC%20TCGA%20Liver%20Cancer%20(LIHC)&removeHub=https%3A%2F%2Fxena.treehouse.gi.ucsc.edu%3A443, https://xenabrowser.net/datapages/?cohort=MET500%20(expression%20centric)&removeHub=https%3A%2F%2Fxena.treehouse.gi.ucsc.edu%3A443).

### 2.3 Techniques used

#### 2.3.1 Multiplexed immunohistochemistry

FFPE blocks were cut into 5 uM sections for all cohorts, except for the MOHCCN-P2 cohort, where sections were 10 uM tick. Tissue sections were stained with a 6-marker panel containing CD3 (Clone 2GV6, Cat. No. 5278422001, Ventana), CD68 (Clone KP1, Cat. No. M0814, Agilent), CD8 (Clone SP57, Cat. No. 5937248001, Ventana), FoxP3 (Clone E7/236, Cat. No. ab20034, Abcam), CD20 (Clone L26, Cat. No. M0755, Agilent), and pan-cytokeratin (PanCK, Clones AE1/AE3, Cat. No. M351501-2, Agilent) antibodies and DAPI for cellular segmentation (Akoya). Melanoma tissues were stained with a melanoma cocktail (MEL, Cat. No. ab733, Abcam) and SOX10 antibody (Clone BC34, Cat. No. BC-ACI3099C Biocare Medical) instead of PanCK. Staining was done on an IP-FLX autostainer (BioCare) with OPAL dyes (Akoya Biosciences Cat# NEL811001KT), following the manufacture’s protocol. Stained slides were scanned using the Vectra 3 imaging system (Akoya Biosciences). Regions of interest were annotated by pathologists (C.W. and H.B.). Image analysis was performed in inForm software v2.6.1. Tissues were segmented into intra-tumoral and stromal regions based on panCK or MEL/SOX10 staining. Cells were classified as CD8+ T cells (CD3+CD8+), CD4+ T cells (CD3+CD8−, CD3+Foxp3+), Treg (CD3+FoxP3+), B cells (CD20+), or macrophages (CD68+). Cell counts were normalized by tissue area and reported as cell/mm^2^.

#### 2.3.2 Bulk RNA-sequencing

Immune cell content was estimated using gene expression values from bulk RNA-sequencing data, with a mean of 118M paired-end reads (range: 24M - 482M). RNA-sequencing fastq files were aligned to the hg38 reference genome and gencode v31 transcriptome reference using STAR (v 2.7.10b). Alignments were then processed using RSEM (v 1.3.3) to generate gene and isoform abundance estimates. Transcripts per million (TPM) normalized gene expression values were processed using xCell2 (v0.99) (19) with default parameters and the PanCancer reference, produced by Nofech-Mozes et al (20). xCell2 is a modern immune cell deconvolution algorithm with reported enhanced accuracy, minimized spillover between closely related cell types, and greater flexibility in reference training, leading to more precise discrimination of complex cellular heterogeneity in tissue samples, as reported by the authors and confirmed through our own observations (21). Differences in cell-type abundance between the two groups were assessed using a likelihood-ratio test of linear mixed effect models (lmm; xCELL2, mIHC) with primary disease site as a random intercept for the primary vs metastasis comparisons, or a Wilcoxon-rank sum test for the head and neck IO vs naive comparisons. Correction for multiple comparisons was performed using the Benjamini-Hochberg (BH) method.

### 2.4 Computational analysis and comparison between techniques

To enable a direct comparison between immune cell subtypes identified by both mIHC and RNA-seq deconvolution a categorical matching system was established (**Supplementary Table 2**). This system aligns the five classes of immune cells identified by immunohistochemistry (IHC) - CD4+ T cells, CD8+ T cells, regulatory T cells (Tregs), B cells, and macrophages - with the immune cell subtype information provided by RNA-seq deconvolution methods. This matching approach enables a more standardized comparison of immune cell populations across the two methodologies, accounting for differences in resolution and classification between mIHC and RNA-seq-based deconvolution.

Correlations between cell type abundances estimated by xCell2 and mIHC were assessed using Spearman’s rank correlation coefficient. This non-parametric measure was chosen to evaluate the strength and direction of the monotonic relationship between the two methods, as it does not assume a linear relationship or normal distribution of the data. The strength of correlations was interpreted based on the magnitude of the correlation coefficient, with values closer to +1 or -1 indicating stronger positive or negative correlations, respectively. Confidence intervals for correlation coefficients were also calculated to provide a measure of precision for the estimated correlations. Correlation coefficients with associated p-values less than 0.05 were considered statistically significant.

## 3 Results

### 3.1 Study population characteristics

**Figure 1:**
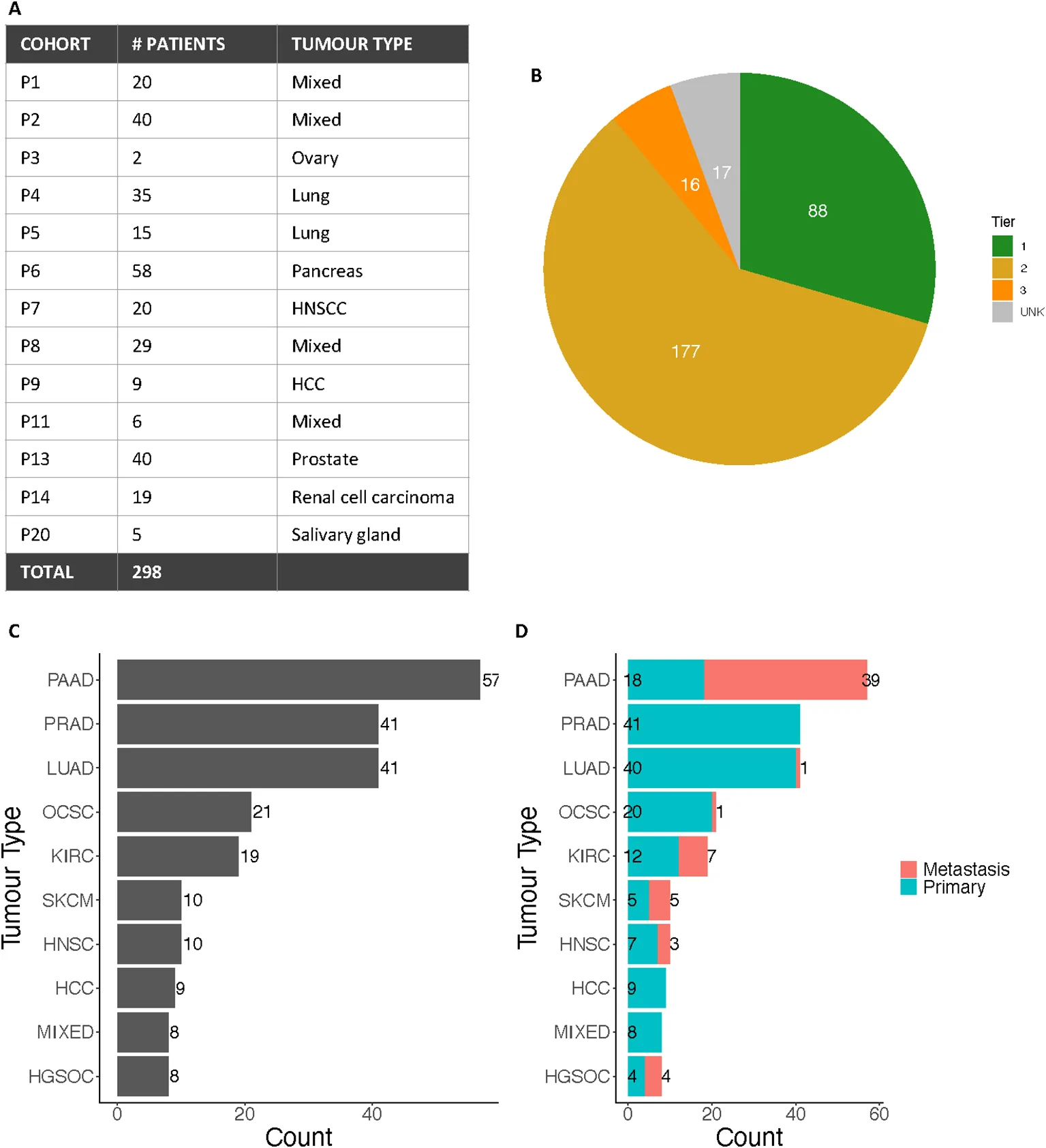
Cohort Summary. **(A)** Number of patients per MOHCNN cohort and tumour type; **(B)** Distribution of paired samples by Tiers; **(C)** Distribution of the 10 more common tumour types/subtypes; **(D)** Distribution of the biopsy site by tumour type/subtype. ONCOTREE codes used in this study correspond to the following tumour types-PAAD, pancreatic adenocarcinoma; PRAD, prostate adenocarcinoma; LUAD, lung adenocarcinoma; OCSC, oral cavity squamous cell carcinoma; KIRC, kidney renal clear cell carcinoma; SKCM, skin cutaneous melanoma; HNSC, head and neck squamous cell carcinoma; HCC, hepatocellular carcinoma; HGSOC, high-grade serous ovarian cancer.

This study analyzed specimens from 298 patients, derived from 13 MOHCCN cohorts, encompassing 20 different tumor types, with all data collected by February 2025. The four most prevalent tumor types in the study population were pancreatic cancer (n=58, 19.5%), lung cancer (n=52, 17.4%), head and neck cancer (n=44, 14.8%), and prostate cancer (n=41, 13.8%), collectively accounting for 65.5% of the samples. A comprehensive breakdown of the study population and samples, categorized by cohort, tumor type, tier, and tissue of origin (primary versus metastatic), is presented in **Figure 1**. Comparisons based on highly matched tissue samples (Tier 1) were conducted using 88 paired samples.

### 3.2 mIHC analysis

**Figure 2:**
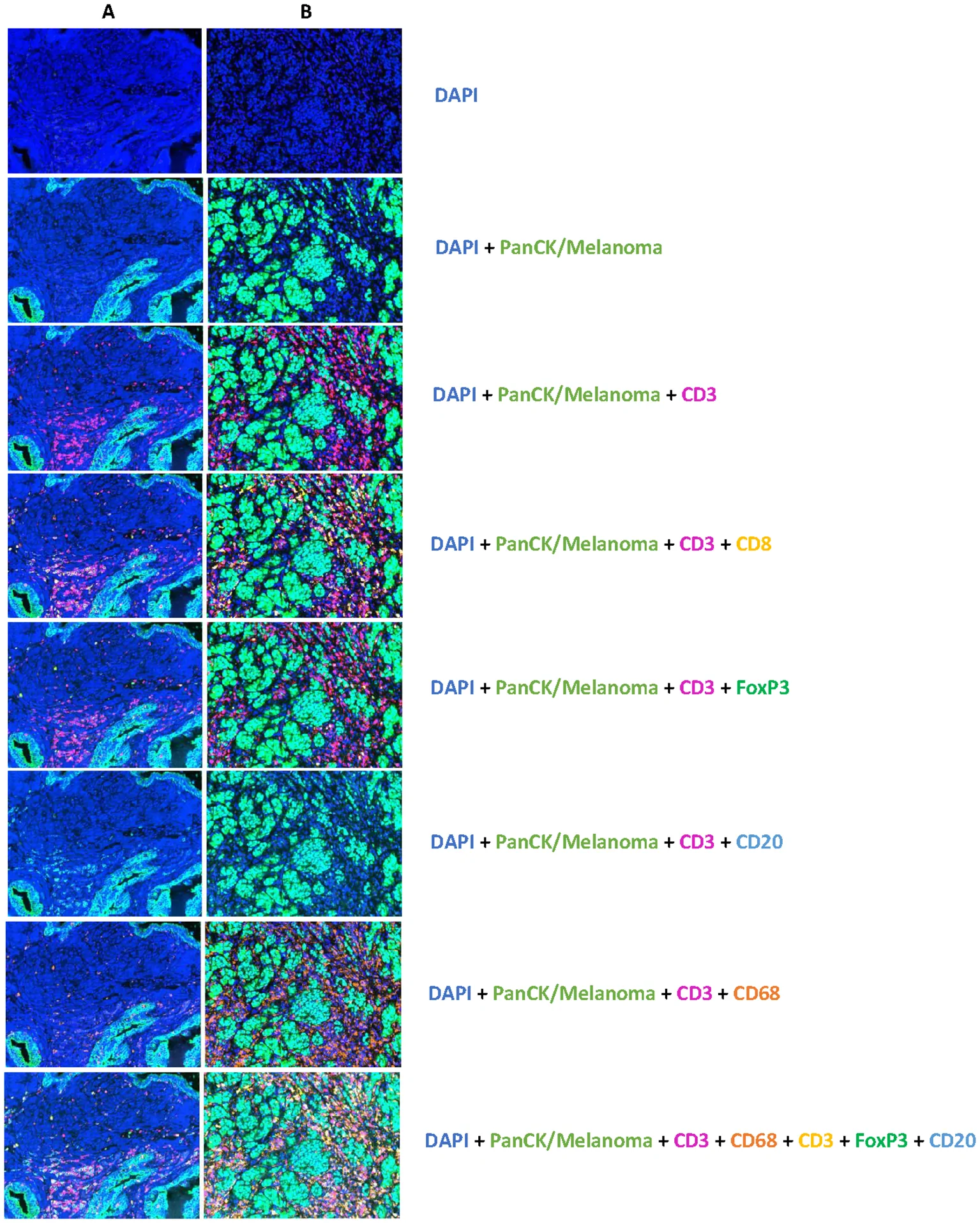
Representative images of multiplexed immunohistochemistry of immune cells with corresponding marker panels in two patient samples. **(A)** Ovarian cancer **(B)** Melanoma.

The mIHC panel was able to detect five different immune cell types: CD4 and CD8 T cells, regulatory T cells (Tregs), B cells and macrophages. An example of a mIHC stained sample from two patients with ovarian cancer and melanoma can be seen in **Figure 2**.

**Figure 3:**
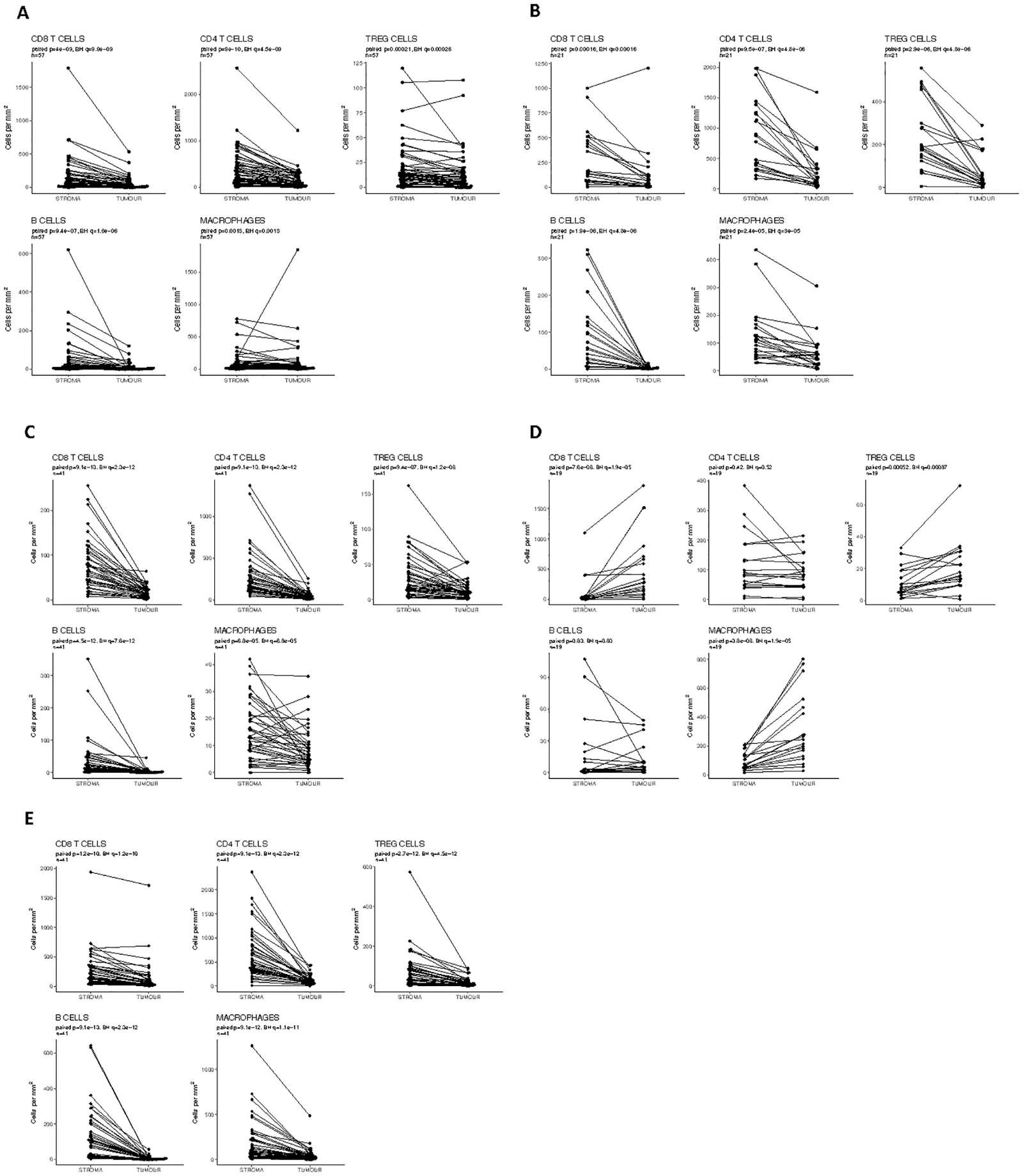
Immune cell density according to tumour region for the five most common tumour types. **(A)** PAAD, pancreatic adenocarcinoma; **(B)** OCSC, oral cavity squamous cell carcinoma; **(C)** PRAD, prostate adenocarcinoma; **(D)** KIRC, kidney renal clear cell carcinoma; **(E)** LUAD, lung adenocarcinoma. Statistical significance (P-values) is indicated above each plot.

Immune cell density was consistently higher in stroma vs. tumor regions for all immune cell types and for the five most prevalent tumor types in our cohort, with the exception for samples from kidney renal clear cell carcinoma (KIRC) where CD8 T cells, Treg cells and macrophages showed higher infiltration in the tumor region (**Figure 3**). Tumor and stroma area were defined by PanCK or melanoma cocktail for carcinoma and melanoma cases respectively.

### 3.3 Analysis of correlations

#### 3.3.1 General correlation between mIHC and xCell2 deconvolution

**Figure 4:**
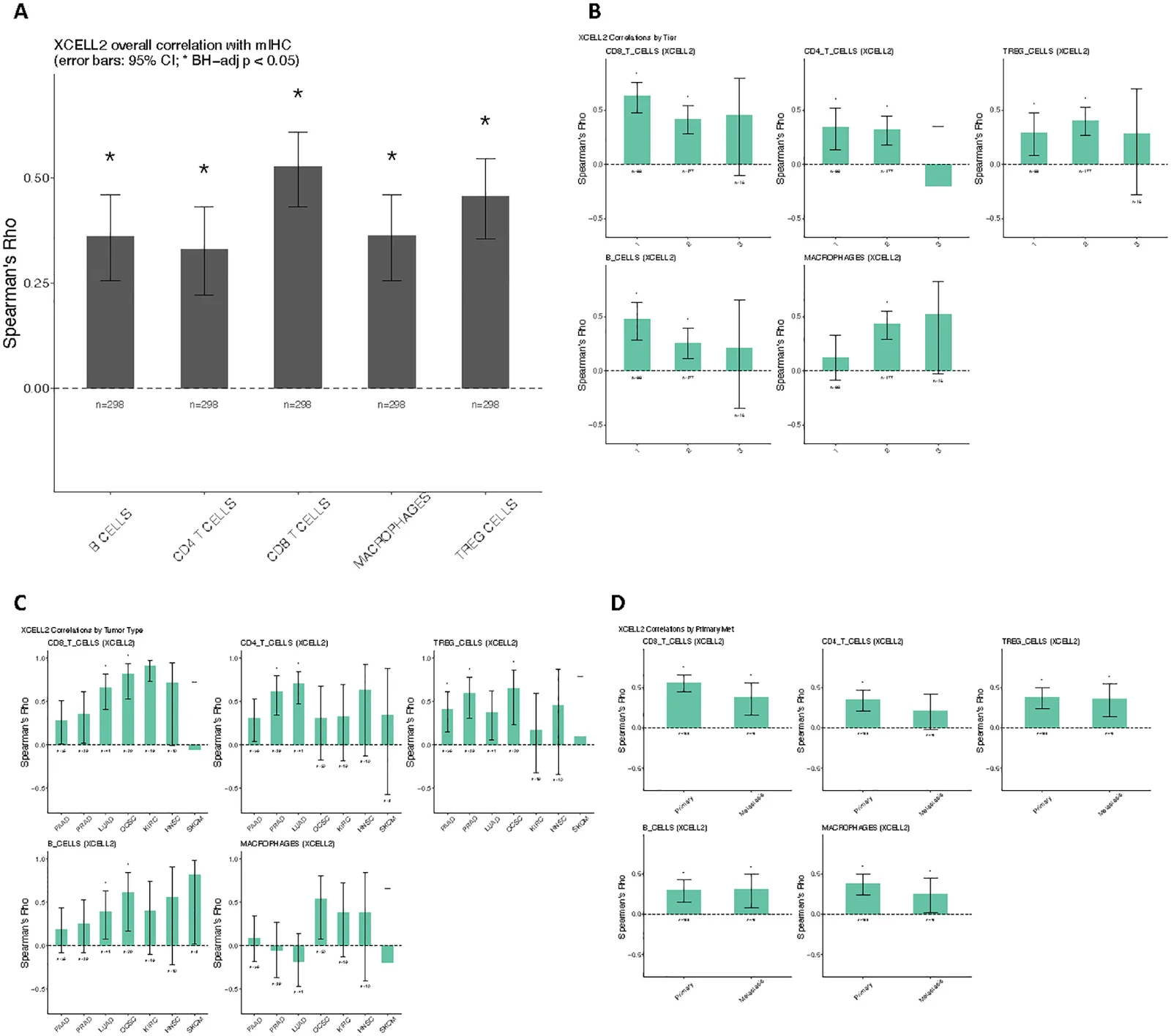
Spearman's rank correlation between mIHC vs. xCell2 deconvolution tool regarding the five immune cell subtypes. **(A)** All samples **(B)** Tier; **(C)** Tumour type (Tiers 1 and 2); **(D)** Biopsy site (Tiers 1 and 2). *statistical significance for the correlation coefficient between mIHC and xCell2 after BH-FDR. ONCOTREE codes correspond to the following tumour types-PAAD, pancreatic adenocarcinoma; PRAD, prostate adenocarcinoma; LUAD, lung adenocarcinoma; OCSC, oral cavity squamous cell carcinoma; KIRC, kidney renal clear cell carcinoma; HNSC, head and neck squamous cell carcinoma; SKCM, skin cutaneous melanoma.

Analysis of all the samples regardless of tumor type, tier and tumor region (stroma/tumor), showed that correlation between mIHC and xCell2 deconvolution was overall modest (Spearman’s rho 0.35 – 0.50) and non-specific for any of the immune cell types, although with a tendency toward better correlation for CD8 T cells (Spearman’s rho 0.50) (**Figure 4A**). Concordance was assessed using partial Spearman correlation, with tumor purity as a covariate.

#### 3.3.2 Tier influence on immune cell type analysis

We performed a separate correlation analysis on samples by Tier. For all 5 immune cell types, only Tiers 1 and 2 (i.e. mIHC and RNA samples taken from the same procedure) showed significant correlation between mIHC and xCell2 derived immune cell density (**Figure 4B**).

#### 3.3.3 Tumor type influence on immune cell type analysis

**Figure 5:**
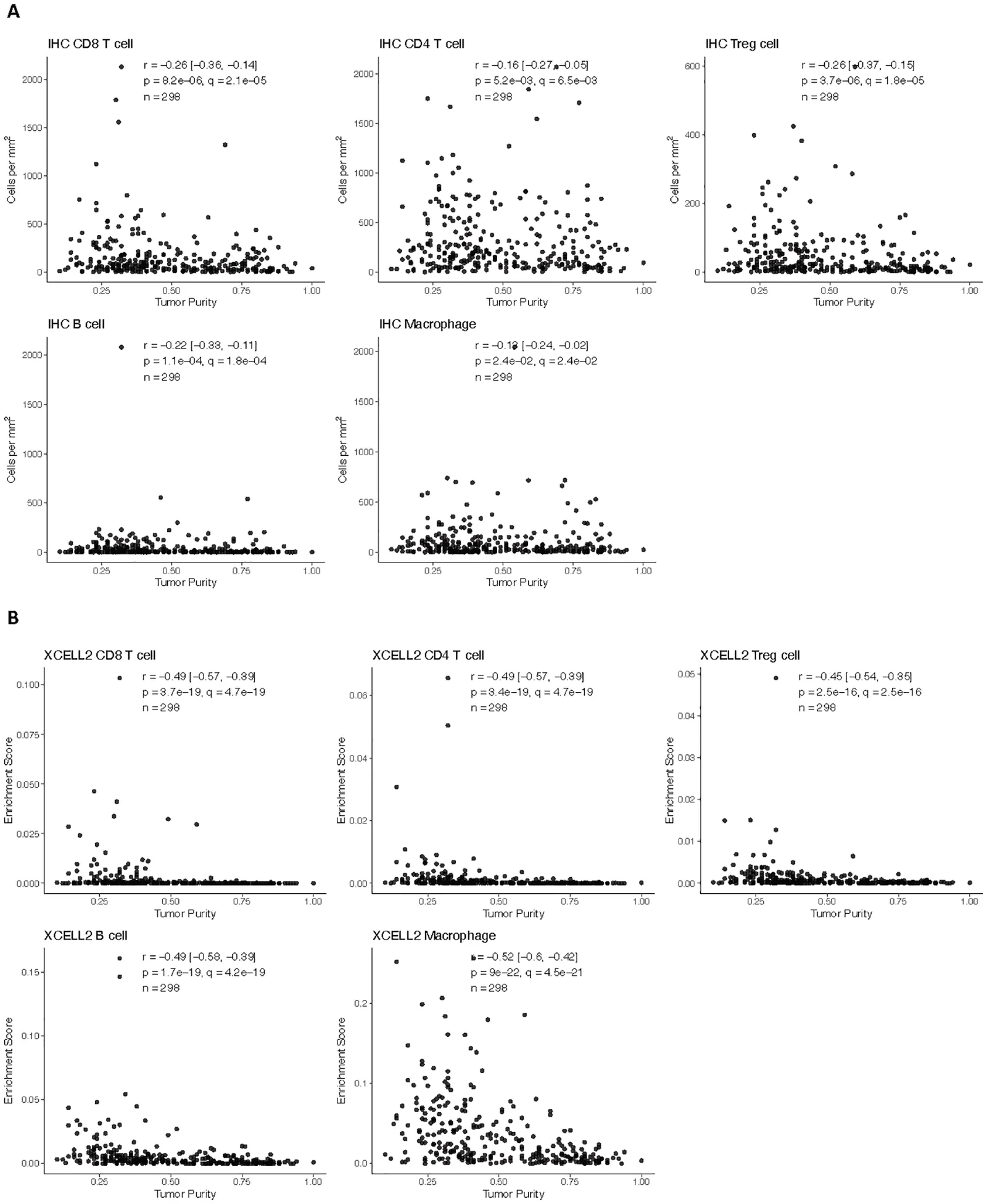
Correlation between tumour purity and immune cell estimates. **(A)** IHC; **(B)** xCell2.

We analyzed the 20 tumor types present in our cohort, with a focus on the five most common cancer types present (pancreas, HNSCC, prostate, renal, lung). Focusing only on Tier 1 and 2 samples, we found that tumor type influences the correlation between mIHC and xCell2 as well as differences between immune cell subtypes (**Figure 4C**). CD8 T cells was the immune cell population with better correlation overall in different tumor types, notably in KIRC, HNSCC in general and also in the subset of OCSC. Correlation for all immune cell types was overall better for HNSCC and worse for pancreatic cancer, with melanoma samples showing a variable correlation dependent on the immune cell type. An analysis of tumor purity across all immune cell types showed a consistent negative correlation for both IHC and xCell2 (**Figure 5**).

#### 3.3.4 Site of biopsy analysis (primary vs. metastatic samples)

When limited to Tier 1 and 2 samples, concordance was marginally higher for all immune cell types in primary vs. metastatic samples (**Figure 4D**). In this analysis, CD8 T cells showed the strongest association (*ρ* = 0.55) in primary samples, while for the other immune cell types the correlation was less evident (*ρ* ∼0.40).

#### 3.3.5 Liver TME in primary hepatocellular carcinoma vs. metastatic liver lesions

**Figure 6:**
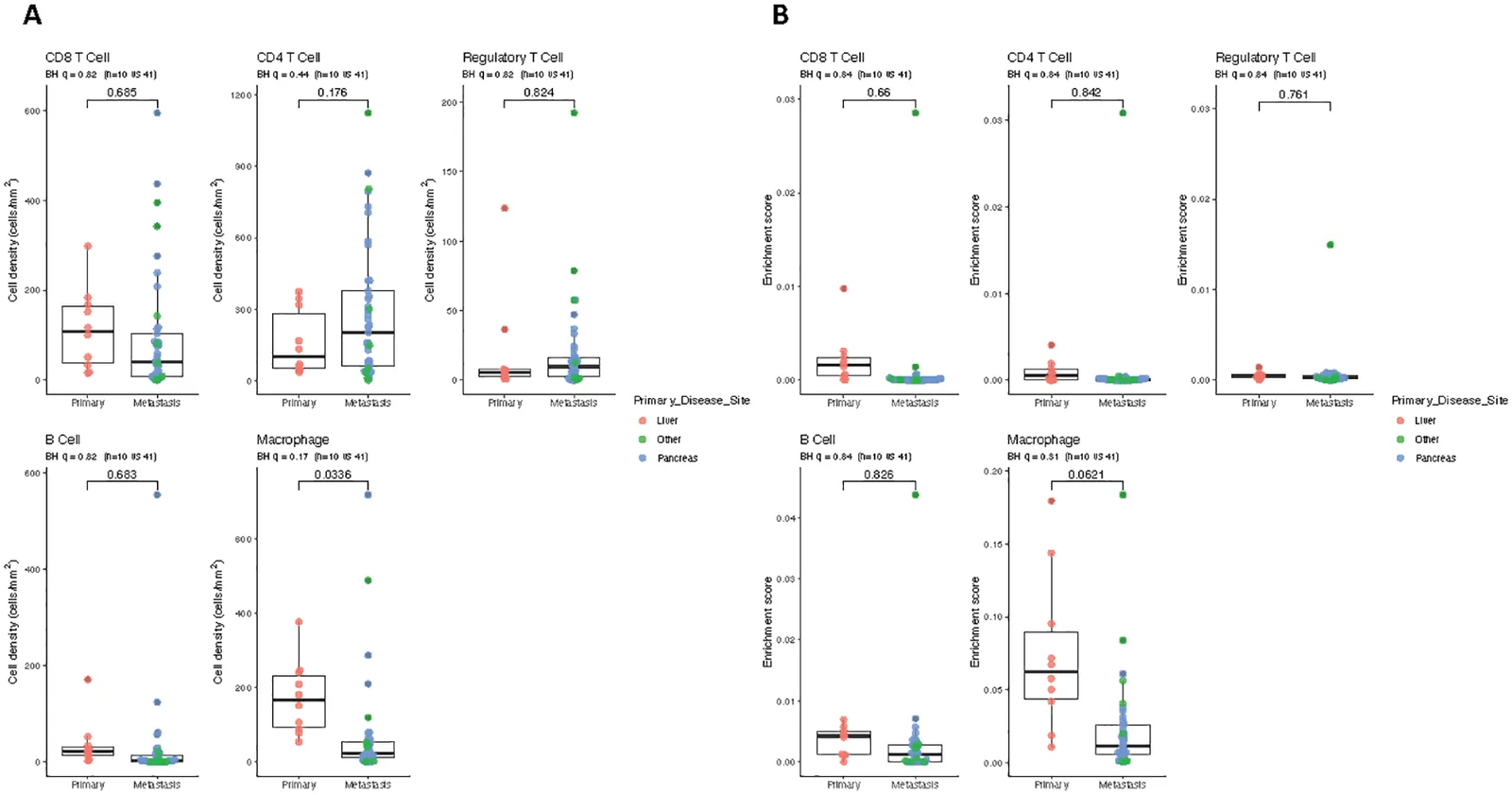
Comparison of five immune cell types between primary liver cancers (hepatocellular carcinoma - HCC). vs. metastatic liver lesions from pancreas primary and other tumour types. **(A)** using multiplexed IHC; **(B)** using xCell2. Statistical significance (P-values) is indicated above each plot.

Liver samples from four cohorts were analyzed to assess the composition of immune cells in the TME, including primary hepatocellular carcinoma (HCC, n = 9) and liver metastases from other tumor types (pancreatic cancer, n = 34; melanoma, n=2; colorectal cancer, n=1; HNSCC, n=1) (**Figure 6**).

Macrophage cell density (mIHC) was observed to be significantly higher in primary HCC samples compared to liver metastases from other sites (p = 0.0336; **Figure 6A**). This difference was also observed in the RNA-seq deconvolution results from xCell2 (p = 0.0621; **Figure 6B**), although not statistically significant. This result was further validated using external datasets MET 500 and TCGA-LIHC (p = 0.00069; **Supplementary Figure 1**).

#### 3.3.6 Immunotherapy exposure effect on HNSCC subpopulation

**Figure 7:**
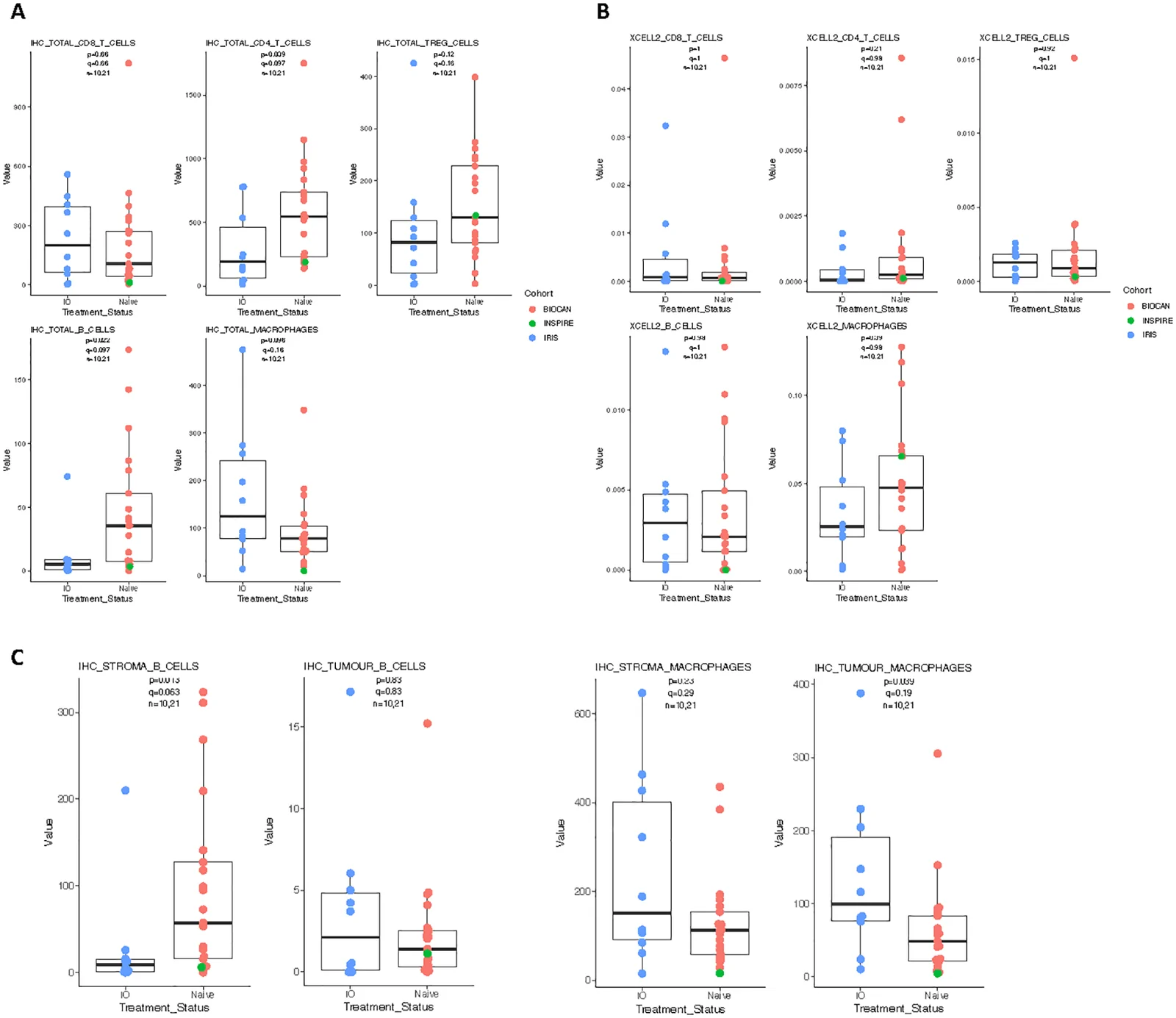
Immunotherapy effect on the five different immune cell types in a sub-cohort of HNSCC patients. **(A)** using mIHC; **(B)** using xCell2; **(C)** mIHC stroma vs. tumour compartment comparison regarding B cells and macrophages. Statistical significance (P-values) is indicated above each plot.

An exploratory analysis was conducted in a subset of patients with HNSCC HPV-negative samples (n= 43) to assess the possible impact of prior immunotherapy in the TME. Samples were stratified into two groups based on prior exposure to IO: immunotherapy-exposed patients (MOHCCN-P8 n=10) and immunotherapy-naïve patients (MOHCCN-P7 n=20, MOHCCN-P1 n=13) (**Figure 7**).

mIHC detected a significantly decreased presence of CD4 T cells (p = 0.039) and B cells (p = 0.022) in IO treated patients, whereas the xCell2 deconvolution method did not identify significant differences in these cell populations between treatment groups. A sub-analysis in this subset of HNSCC samples with a focus on the different tumor compartments (stromal vs. tumor) using only mIHC data showed that B cells were significantly lower in the stroma of IO treated patients (p = 0.013) whereas intra-tumor macrophages were significantly higher in IO-exposed patients (p = 0.039).

## 4 Discussion

### 4.1 Sample heterogeneity plays a role in mIHC vs. RNA-seq correlation​, with Tier 1–2 samples with better correlation between methods

Our results highlight the importance of proper annotation when performing correlative analysis. When all samples were grouped, the overall correlation between mIHC and xCell2 was modest, but assigning samples to Tiers, especially Tier 1 and 2 where matched tissues were used, increased concordance. Sample heterogeneity and technical variability weaken RNA-seq reproducibility compared to mIHC (22). Tier classification helped reduce variability and improve reliability. Future studies with standardized sampling, larger cohorts, and a tighter focus on specific tumor types will further strengthen statistical power and method concordance.

Previously published work comparing clinically actionable biomarker genes using RNA sequencing and IHC-measured expression profiles (17) showed high and statistically significant correlations, but for immune cell profiling few comparisons have been done so far. For example, Wu M et al. (23) developed a TME score for cervical cancer recurrence using RNA-seq tools (24) and validated results with mIHC, but no direct correlation analysis between the two assays was performed. Similarly, in melanoma (25), multiomic analysis using whole genome and transcriptome sequencing (WGTS) and IHC confirmed the complementary information provided by mIHC and RNA-seq, with mIHC providing spatial and phenotypic conclusions, while RNA-seq offering a molecular and functional insight.

In our study, a tendency for better correlation between mIHC and RNA-seq data was observed with CD8+ T cells were observed in tumor types like KIRC and OCSC, likely due to their known prominent immune infiltration and heterogeneity. We hypothesize that this abundance and diversity of immune cells make it easier for both mIHC and RNA-seq tools to capture and quantify immune cell subsets, increasing the likelihood of concordant results. Additionally, the fundamental difference that mIHC evaluates protein expression whereas RNA-seq measures gene expression may serve as a general underlying explanation for some of the discordant results observed between the two techniques regarding immune cell profiling.

### 4.2 Stromal compartment has higher immune cell infiltration when compared to tumor compartment in the TME

Our study found that immune cell infiltration is generally higher in the stromal compartment compared to the tumor compartment across several cancer types, including pancreatic adenocarcinoma (PAAD), prostate adenocarcinoma (PRAD), OCSC, and lung adenocarcinoma (LUAD) cancers. This observation is consistent with previous reports demonstrating that the stroma often serves as the primary site for immune cell accumulation within the TME, acting as a barrier and a site of active immune-tumor cell interactions (26). For example, in PAAD, immune cells such as T cells and macrophages are predominantly localized in the stroma, with minimal infiltration into the tumor nests themselves, a pattern also observed in PRAD and OCSC. Similarly, spatial analyses in LUAD have emphasized the distinct immune landscapes between stromal and tumor compartments, with stromal regions typically displaying greater immune cell density (27).

Interestingly, our data revealed an exception in KIRC, where immune cell infiltration was higher within the tumor compartment rather than the stroma. This divergence may reflect unique aspects of the renal TME, including its vascularity and immune cell recruitment patterns, which can differ markedly from other solid tumors (28). These findings underscore the importance of considering compartmental localization when evaluating immune infiltration and underscores the heterogeneity of tumor-immune interactions across cancer types.

### 4.3 Tumor type determines mIHC vs. xCell2 correlation and immune cell types influences this association

Our analysis included 20 tumor types, though several had few samples. The most common tumors showed variable agreement between profiling methods: OCSC had strong concordance, with both assays identifying similar immune populations, consistent with prior studies in less complex stromal architecture tumors and lower levels of cellular heterogeneity (17, 29). In contrast, PAAD showed lower agreement, likely due to their dense stroma and low immune infiltration, which complicates both spatial and gene expression analyses (30).

Surprisingly, correlation between mIHC and RNA-seq in melanoma and LUAD was lower than expected, despite their high immune infiltration. Both cancers are highly immunogenic, but they also display considerable immune heterogeneity and complex TMEs (31). This variability introduces technical and biological differences in what each method detects, reducing assay concordance (32). Sampling variation, regional immune cell distribution, and differences in tumor purity further contribute to discrepancies between molecular and histological assessments, as supported by literature in these cancer types (33).

When examining specific immune cell populations, concordance between mIHC and RNA-seq was strongest for CD8^+^ T cells, with a great number of tumor types exhibiting correlation coefficients above 0.7. Several factors may help explain this pattern of concordance. First, CD8^+^ T cells have highly specific markers (CD8A and CD8B) that are reliably detected at both the protein and mRNA levels (34). Additionally, CD8^+^ tumor-infiltrating lymphocytes (TILs) often tend to occur at higher frequencies within the TME and can form clonally expanded populations, thereby increasing their detectability and quantification accuracy across both platforms (35). Furthermore, compared to other immune cell types, CD8^+^ T cells tend to display reduced phenotypic and functional heterogeneity within tumors. This relative homogeneity likely contributes to the consistency observed between protein-based and transcriptomic measurements in this dataset.

Collectively, these results draws attention to the need for careful interpretation of immune profiling data and suggest that the choice of methodology and tumor context are critical considerations for accurate characterization of the immune landscape. Our findings underscore the importance of tumor-specific factors—such as tissue composition, immune cell localization, and sample purity—in shaping the reliability of immune cell quantification across different platforms.

### 4.4 Site of biopsy may influence the correlation, with better correlation seen in primary samples vs. metastatic

In our study, we observed that the correlation between immune cell type estimates obtained by mIHC and RNA-seq–based deconvolution methods was stronger in primary tumor samples compared to metastatic samples. This finding is consistent with prior literature demonstrating that immune cell profiling in primary tumors tends to be more robust and reproducible, likely due to the more defined, spatially organized and less heterogeneous immune microenvironment present at the primary site (36). Additionally, technical and biological factors such as tumor evolution, prior treatments, and microenvironmental remodeling in metastatic lesions may further contribute to discrepancies between both methods (36). This observation shows the importance of tumor context when interpreting immune profiling data and suggests that concordance between different analytical platforms may be highest in primary tumor settings.

### 4.5 Macrophages in liver differs between primary HCC cancer vs. metastatic liver deposits

While healthy liver is rich in Kupffer cells, which maintain immune tolerance through an anti-inflammatory phenotype, in hepatocellular carcinoma (HCC) the TME is dominated by tumor-associated macrophages (TAMs) predominantly exhibiting an M2-like, immunosuppressive phenotype (37). In contrast, liver metastases show greater macrophage heterogeneity (38), and Kupffer cells may adopt a pro-inflammatory (M1) phenotype, supporting tumor growth (38). Interestingly, in our cohort, macrophages were the only immune cell type showing a statistically significant difference between HCC and metastatic liver deposits but only when assessed by mIHC, although with a tendency toward the same result when using xCell2. Macrophage density was higher in primary HCC tissue, while metastatic liver lesions, mostly from PAAD, exhibited lower infiltration. Notably, mIHC relies on CD68, a pan-macrophage marker that cannot distinguish between M1 and M2 subtypes, limiting polarization analysis and creating a phenotypic mismatch. Therefore, correlation analysis between these two modalities should be interpreted as reflecting only a partial overlap between total macrophage counts and inferred functional macrophage states, rather than strict biological equivalence. This limitation may partly explain the variability in concordance between the two assays.

It is known that CD68+ macrophages cluster at the invasive front of HCC and correlate with tumor progression, whereas metastatic tumors display distinct spatial and polarization profiles influenced by the more complex metastatic microenvironment (39), emphasizing the role of mIHC in the spatial profiling of the TME. Although detailed polarization could not be assessed, our results also align with prior studies and confirm reliable characterization of our dataset by both mIHC and xCell2 approaches, with differences in macrophage infiltration possibly reflecting the biological context and aggressiveness of the primary tumors (40).

### 4.6 Immunotherapy exposure showed inconsistent impact on the immune cell profile within the HNSCC subpopulation

In the exploratory analysis of the subset of 43 patients with HNSCC analyzed, predominantly comprising OCSC HPV-negative, and stratified into two groups based on prior exposure to IO, the mIHC analysis revealed significant differences in the densities of CD4+ T cells and B cells, while xCell2 method found a similar trend but not statistically significant. The results of this exploratory analysis should be interpreted cautiously in light of the relatively small sample size and the presence of potential confounding variables that were not controlled for, including HPV status, biopsy timing, tumor site, disease stage, and prior treatment history.

Discordances between mIHC and deconvolution methods for immune cell analysis are well-documented and result from both technical and biological differences. mIHC provides direct spatial localization and phenotyping of immune cells within the TME (41), but is limited by antibody cross-reactivity, signal saturation, and complexity of multiplex panels (42). In contrast, xCell2 which infers immune cell proportions from bulk gene expression data, enables high-throughput analysis with less tissue needed, but lacks spatial context and direct cell identification may be limited by overlapping gene expression profiles among related cell types, potentially reducing resolution and resulting in discrepancies when compared to mIHC-derived data and affect accuracy (19, 43).

Our data revealed reduced CD4+ T cells in patients with prior IO, reflecting known immunotherapy-induced changes in the TME. CD4+ T cells promote antitumor responses and their higher levels are linked to better prognosis in HNSCC (44), but IO may deplete or alter these populations through immune editing or exhaustion (45). Compared to tumor-infiltrating T cells, studies of B cells in HNSCC have been less consistent, mainly focusing on baseline status and prognostic significance of B cells in samples treatment naïve, with HPV-positive cancers typically showing higher B cell content (46). Of note, our cohort of HNSCC was mainly composed by OCSC and all were HPV-negative patients. Notably, the observation of higher B cell infiltration in immunotherapy-naïve patients in our cohort is consistent with existing evidence that increased pre-treatment B cell density in HNSCC is associated with prolonged progression-free survival and improved response to PD-1-based immunotherapy (47).

These observations underscore the importance of considering clinical characteristics and treatment history when interpreting immune profiling data, focusing the need for further research to elucidate the mechanisms underlying immunotherapy-associated alterations in immune cell profile dynamic changes.

### 4.7 Selection of mIHC and/or xCell2 for immune cell profiling should be guided by study objectives and expected outcomes

Our study pinpoints important methodological differences in the estimation of immune cell estimation within the TME, though overall concordance between assays improved when technical variables were addressed. Key limitations include the retrospective design, lack of control over Tier selection, and the inclusion of diverse tumor types, which broadened scope but reduced specificity for individual cancers. Differences in disease stage and site also added heterogeneity, impacting immune landscape analysis. Taken together, these factors underscore the need for carefully designed, prospective studies to further refine and validate the methodological approaches for immune profiling in oncology.

## 5 Conclusions

Our work highlights that the observed discordance between mIHC and xCell2 methods reflects fundamental methodological differences, namely, the direct spatial and phenotypic resolution provided by mIHC versus the indirect inference of cell populations from bulk RNA sequencing. This underscores the value of integrating both approaches to achieve a comprehensive and accurate assessment of immune cell populations within the TME. Their combined use is increasingly recognized as best practice for accurate immune cell profiling in complex tissues.

Looking forward, we hypothesize that future prospective studies employing standardized tissue sampling, larger patient cohorts, and a focused analysis of fewer tumor types will enhance statistical power and further improve the correlation between these methodologies. Collectively, our results emphasize that methodological rigor and careful consideration of tumor context are essential for the accurate interpretation of immune profiling data, ultimately advancing our understanding of the tumor-immune landscape in oncology research.

## Data Availability

The clinical and genomic datasets supporting the findings of this study were generated under the Marathon of Hope Cancer Centres Network (MOHCCN). Individual participant-level datasets including raw transcriptome sequencing reads and clinical data are classified as protected and are maintained under a controlled-access tier to safeguard participant privacy. In accordance with the MOHCCN Data Access and Use Policy, these controlled-access data are available to qualified researchers. To request access, researchers must submit a formal Data Access Request Form to the MOHCCN Data Access Committee (DAC) Secretariat via email at mohdatarequests@tfri.ca.

## References

[1] Anderson NM Simon MC . The tumor microenvironment. Curr Biol. (2020) 30:R921–5. doi: 10.1016/j.cub.2020.06.081 32810447 PMC8194051

[2] Peña-Romero AC Orenes-Piñero E . Dual effect of immune cells within tumour microenvironment: Pro- and anti-tumour effects and their triggers. Cancers (Basel). (2022) 14. doi: 10.3390/cancers14071681 35406451 PMC8996887

[3] Fridman WH Zitvogel L Sautès–Fridman C Kroemer G . The immune contexture in cancer prognosis and treatment. Nat Rev Clin Oncol. (2017) 14:717–34. doi: 10.1038/nrclinonc.2017.101 28741618

[4] Chen B Li H Liu C Xiang X Wang S Wu A . Prognostic value of the common tumour-infiltrating lymphocyte subtypes for patients with non-small cell lung cancer: A meta-analysis. PloS One. (2020) 15:e0242173. doi: 10.1371/journal.pone.0242173 33170901 PMC7654825

[5] Lu C Gao Z Wu D Zheng J Hu C Huang D . Understanding the dynamics of TKI-induced changes in the tumor immune microenvironment for improved therapeutic effect. J Immunother Cancer. (2024) 12. doi: 10.1136/jitc-2024-009165 38908857 PMC11328648

[6] Väyrynen JP Lau MC Haruki K Väyrynen SA Dias Costa A Borowsky J . Prognostic significance of immune cell populations identified by machine learning in colorectal cancer using routine hematoxylin and eosin-stained sections. Clin Cancer Res. (2020) 26:4326–38. doi: 10.1158/1078-0432.CCR-20-0071 PMC7442724 32439699

[7] Edin S Kaprio T Hagström J Larsson P Mustonen H Böckelman C . The prognostic importance of CD20+ B lymphocytes in colorectal cancer and the relation to other immune cell subsets. Sci Rep. (2019) 9:19997. doi: 10.1038/s41598-019-56441-8 31882709 PMC6934737

[8] Väyrynen JP Haruki K Väyrynen SA Lau MC Dias Costa A Borowsky J . Prognostic significance of myeloid immune cells and their spatial distribution in the colorectal cancer microenvironment. J Immunother Cancer. (2021) 9:e002297. doi: 10.1136/jitc-2020-002297 33931472 PMC8098931

[9] Galon J Costes A Sanchez-Cabo F Kirilovsky A Mlecnik B Lagorce-Pagès C . Type, density, and location of immune cells within human colorectal tumors predict clinical outcome. Science. (2006) 313:1960–4. doi: 10.1126/science.1129139 17008531

[10] Chen R Li Q Xu S Ye C Tian T Jiang Q . Modulation of the tumour microenvironment in hepatocellular carcinoma by tyrosine kinase inhibitors: From modulation to combination therapy targeting the microenvironment. Cancer Cell Int. (2022) 22:73. doi: 10.1186/s12935-021-02435-4 35148789 PMC8840552

[11] Rojas LA Sethna Z Soares KC Olcese C Pang N Patterson E . Personalized RNA neoantigen vaccines stimulate T cells in pancreatic cancer. Nature. (2023) 618:144–50. doi: 10.1038/s41586-023-06063-y 37165196 PMC10171177

[12] Fava Gaspar C Park JC Falchook GS Wesolowski R Soliman H Ott PA . 658P First-in-human phase I trial of oncolytic herpes simplex virus ONCR-177 alone or in combination with pembrolizumab in advanced solid tumors. Ann Oncol. (2024) 35:S519. doi: 10.1016/j.annonc.2024.08.724 38826717

[13] Hammers HJ Vonmerveldt D Ahn C Nadal RM Drake CG Folkert MR . Combination of dual immune checkpoint inhibition (ICI) with stereotactic radiation (SBRT) in metastatic renal cell carcinoma (mRCC) (RADVAX RCC). J Clin Oncol. (2020) 38:614. doi: 10.1200/jco.2020.38.6_suppl.614

[14] Lopes CDH Braganca Xavier C Torrado C Veneziani AC Megid TBC . A comprehensive exploration of agents targeting tumor microenvironment: Challenges and future perspectives. J Immunother Precis Oncol. (2024) 7:283–99. doi: 10.36401/jipo-24-23 39524466 PMC11541921

[15] Chesney J Puzanov I Collichio F Singh P Milhem MM Glaspy J . Randomized, open-label phase II study evaluating the efficacy and safety of talimogene laherparepvec in combination with ipilimumab versus ipilimumab alone in patients with advanced, unresectable melanoma. J Clin Oncol. (2018) 36:1658–67. doi: 10.1200/jco.2017.73.7379 28981385 PMC6075852

[16] Liu L Zhao Q Cheng C Yi J Sun H Wang Q . Analysis of bulk RNA sequencing data reveals novel transcription factors associated with immune infiltration among multiple cancers. Front Immunol. (2021) 12:644350. doi: 10.3389/fimmu.2021.644350 34489925 PMC8417605

[17] Sorokin M Ignatev K Poddubskaya E Vladimirova U Gaifullin N Lantsov D . RNA sequencing in comparison to immunohistochemistry for measuring cancer biomarkers in breast cancer and lung cancer specimens. Biomedicines. (2020) 8. doi: 10.3390/biomedicines8050114 32397474 PMC7277916

[18] Saito N Sato Y Abe H Wada I Kobayashi Y Nagaoka K . Selection of RNA-based evaluation methods for tumor microenvironment by comparing with histochemical and flow cytometric analyses in gastric cancer. Sci Rep. (2022) 12:8576. doi: 10.1038/s41598-022-12610-w 35595859 PMC9122932

[19] Angel A Naom L Nabet-Levy S Aran D . xCell 2.0: Robust algorithm for cell type proportion estimation predicts response to immune checkpoint blockade. bioRxiv. (2024), 2024.09.06.611424. doi: 10.1101/2024.09.06.611424 41044684 PMC12492622

[20] Nofech-Mozes I Soave D Awadalla P Abelson S . Pan-cancer classification of single cells in the tumour microenvironment. Nat Commun. (2023) 14:1615. doi: 10.1038/s41467-023-37353-8 36959212 PMC10036554

[21] Angel A Naom L Nabet S Aran D . xCell 2.0: Robust algorithm for cell type proportion estimation predicts response to immune checkpoint blockade. (2024). doi: 10.1101/2024.09.06.611424 PMC12492622 41044684

[22] Degen PM Medo M . Replicability of bulk RNA-seq differential expression and enrichment analysis results for small cohort sizes. PloS Comput Biol. (2025) 21:e1011630. doi: 10.1371/journal.pcbi.1011630 40324149 PMC12077797

[23] Wu M Li B Shi L Yang L Liang C Wang T . RNA sequencing and multiplexed immunohistochemistry reveal the factors for postoperative recurrence of stage IB-IIA cervical squamous cell carcinoma. Discov Oncol. (2024) 15:422. doi: 10.1007/s12672-024-01283-8 39254825 PMC11387578

[24] Li T Fu J Zeng Z Cohen D Li J Chen Q . TIMER2.0 for analysis of tumor-infiltrating immune cells. Nucleic Acids Res. (2020) 48:W509–14. doi: 10.1093/nar/gkaa407 32442275 PMC7319575

[25] Gide TN Mao Y Scolyer RA Long GV Wilmott JS . Tissue-based profiling techniques to achieve precision medicine in cancer: Opportunities and challenges in melanoma. Clin Cancer Res. (2024) 30:5270–80. doi: 10.1158/1078-0432.ccr-24-1109 39373690

[26] Yoshihara K Shahmoradgoli M Martínez E Vegesna R Kim H Torres-Garcia W . Inferring tumour purity and stromal and immune cell admixture from expression data. Nat Commun. (2013) 4:2612. doi: 10.1038/ncomms3612 24113773 PMC3826632

[27] Page DB Broeckx G Jahangir CA Verbandt S Gupta RR Thagaard J . Spatial analyses of immune cell infiltration in cancer: Current methods and future directions: A report of the International Immuno-Oncology Biomarker Working Group on Breast Cancer. J Pathol. (2023) 260:514–32. doi: 10.1136/bmj.1.3973.472 PMC11288334 37608771

[28] Vuong L Kotecha RR Voss MH Hakimi AA . Tumor microenvironment dynamics in clear-cell renal cell carcinoma. Cancer Discov. (2019) 9:1349–57. doi: 10.1158/2159-8290.cd-19-0499 31527133 PMC6774890

[29] Kim W-J Choi BR Noh JJ Lee Y-Y Kim T-J Lee J-W . Comparison of RNA-seq and microarray in the prediction of protein expression and survival prediction. Front Genet. (2024) 15. doi: 10.3389/fgene.2024.1342021 38463169 PMC10920353

[30] Chen K Wang Q Liu X Tian X Dong A Yang Y . Immune profiling and prognostic model of pancreatic cancer using quantitative pathology and single-cell RNA sequencing. J Transl Med. (2023) 21:210. doi: 10.1186/s12967-023-04051-4 36944944 PMC10031915

[31] Izar B Jerby-Arnon L Rotem A Shah P Liu D Zhang G . Single-cell RNA-sequencing and -imaging of melanoma ecosystems reveals sources of resistance to immune checkpoint blockade. J Clin Oncol. (2018) 36:3074. doi: 10.1200/jco.2018.36.15_suppl.3074 42148471

[32] Zhao W Zhu B Hutchinson A Pesatori AC Consonni D Caporaso NE . Clinical implications of inter- and intratumor heterogeneity of immune cell markers in lung cancer. J Natl Cancer Inst. (2022) 114:280–9. doi: 10.1093/jnci/djab157 34402912 PMC8826638

[33] Aran D Sirota M Butte AJ . Systematic pan-cancer analysis of tumour purity. Nat Commun. (2015) 6:8971. doi: 10.1038/ncomms9971 26634437 PMC4671203

[34] Saleh R Sasidharan Nair V Toor SM Taha RZ Murshed K Al-Dhaheri M . Differential gene expression of tumor-infiltrating CD8(+) T cells in advanced versus early-stage colorectal cancer and identification of a gene signature of poor prognosis. J Immunother Cancer. (2020) 8. doi: 10.1136/jitc-2020-001294 32948653 PMC7511623

[35] Komuro H Shinohara S Fukushima Y Demachi-Okamura A Muraoka D Masago K . Single-cell sequencing on CD8(+) TILs revealed the nature of exhausted T cells recognizing neoantigen and cancer/testis antigen in non-small cell lung cancer. J Immunother Cancer. (2023) 11. doi: 10.1136/jitc-2023-007180 37544663 PMC10407349

[36] Banik G Betts CB Liudahl SM Sivagnanam S Kawashima R Cotechini T . High-dimensional multiplexed immunohistochemical characterization of immune contexture in human cancers. Methods Enzymol. (2020) 635:1–20. doi: 10.1016/bs.mie.2019.05.039 32122539 PMC7390987

[37] Quaranta V Ballarò C Giannelli G . Macrophages orchestrate the liver tumor microenvironment. Cancers (Basel). (2024) 16. doi: 10.3390/cancers16091772 38730724 PMC11083142

[38] Yuan Q Jia L Yang J Li W . The role of macrophages in liver metastasis: Mechanisms and therapeutic prospects. Front Immunol. (2025) 16. doi: 10.3389/fimmu.2025.1542197 40034694 PMC11872939

[39] Avădănei ER Wierzbicki PM Giuşcă SE Grigoraş A Amălinei C Căruntu ID . Macrophage profile in primary versus secondary liver tumors. Folia Histochem Cytobiol. (2014) 52:112–23. doi: 10.5603/FHC.2014.0014 25007179

[40] Sathe A Mason K Grimes SM Zhou Z Lau BT Bai X . Colorectal cancer metastases in the liver establish immunosuppressive spatial networking between tumor-associated SPP1+ macrophages and fibroblasts. Clin Cancer Res. (2023) 29:244–60. doi: 10.1158/1078-0432.ccr-22-2041 36239989 PMC9811165

[41] Giesen C Wang HAO Schapiro D Zivanovic N Jacobs A Hattendorf B . Highly multiplexed imaging of tumor tissues with subcellular resolution by mass cytometry. Nat Methods. (2014) 11:417–22. doi: 10.1038/nmeth.2869 24584193

[42] Tsujikawa T Kumar S Borkar RN Azimi V Thibault G Chang YH . Quantitative multiplex immunohistochemistry reveals myeloid-inflamed tumor-immune complexity associated with poor prognosis. Cell Rep. (2017) 19:203–17. doi: 10.1016/j.celrep.2017.03.037 28380359 PMC5564306

[43] Newman AM Liu CL Green MR Gentles AJ Feng W Xu Y . Robust enumeration of cell subsets from tissue expression profiles. Nat Methods. (2015) 12:453–7. doi: 10.1038/nmeth.3337 25822800 PMC4739640

[44] Badoual C Hans S Rodriguez J Peyrard S Klein C Agueznay Nel H . Prognostic value of tumor-infiltrating CD4+ T-cell subpopulations in head and neck cancers. Clin Cancer Res. (2006) 12:465–72. doi: 10.1158/1078-0432.ccr-05-1886 16428488

[45] Damasio MPS Nascimento CS Andrade LM de Oliveira VL Calzavara-Silva CE . The role of T-cells in head and neck squamous cell carcinoma: From immunity to immunotherapy. Front Oncol. (2022) 12:1021609. doi: 10.3389/fonc.2022.1021609 36338731 PMC9632296

[46] Wang Z Wang Q Tao Y Chen J Yuan Z Wang P . Characterization of immune microenvironment in patients with HPV-positive and negative head and neck cancer. Sci Data. (2023) 10:694. doi: 10.1038/s41597-023-02611-3 37828063 PMC10570276

[47] Gavrielatou N Fortis E Spathis A Anastasiou M Economopoulou P Foukas GRP . B-cell infiltration is associated with survival outcomes following programmed cell death protein 1 inhibition in head and neck squamous cell carcinoma. Ann Oncol. (2024) 35:340–50. doi: 10.2139/ssrn.4574783 38159908

