# Supplementary material for: Immune profiling by bulk RNA-seq and multiplex immunofluorescence imaging across multiple cancer types
Source: Front Oncol. 2026 Aug 6;16:1876853. doi: 10.3389/fonc.2026.1876853 (PMC13489921; doi:10.3389/fonc.2026.1876853)
Supplement: Supplementary file 1 [file DataSheet1.pdf]

Supplementary table 1 - Detailed description of cohorts from MOHCNN used in this project

MOHCCN Cohorts	Description of Cohort
**P1**	Patients within 5 cohorts treated with single agent pembrolizumab: **(A) squamous cell carcinoma of the head and neck, (B) triple negative breast cancer, (C) high grade serous ovarian cancer, (D) melanoma and (E) mixed solid tumors.** It comprises pre-treatment and on-treatment tumor and blood samples, evaluating biomarkers of sensitivity/resistance to immunotherapy.
**P2**	Patients with **advanced solid tumors** whose archival tumor tissue was genetically tested on NGS technology, informing the use of approved therapies to facilitate targeted therapy and immune therapy clinical trials in Ontario.
**P3**	Patients with **high-grade serous ovarian cancer**, evaluating the biology of this disease, from diagnosis throughout disease course, focusing on genomic and immune signature profiles.
**P4**	Patients with **lung adenocarcinoma**, lifetime never smokers or IO-treated, who have resected or large biopsy samples with available fresh-frozen tissue and FFPE tissue, evaluating genomic and transcriptomic alterations.
**P5**	Patients with **lung cancer** whose tumour (early-stage resectable or metastatic) have been used to establish patient-derived xenograft (PDX) models and/or organoid cell lines.
**P6**	Patients with **advanced pancreatic cancer**, evaluating the use of whole genome and RNA sequencing for a clinically meaningful turnaround time.
**P7**	Patients with **oral cavity squamous cell cancer**, evaluating clinic-pathological and molecular markers to correlate with prognostic outcomes and to develop genomic biomarkers that can enhance clinical decision-making.
**P8**	Patients with **advanced solid tumors** previously treated with immunotherapy, evaluating genomic, transcriptomic, epigenetic and immunophenotypic profile of primary vs. acquired resistance to anti-PD1/PD-L1 based therapy.
**P9**	Patients with **hepatocellular carcinoma**, evaluating validity and utility of genomics for real-time decision making and investigating the temporal heterogeneity of HCC tumors.
**P11**	Patients with **hereditary cancer syndrome**, evaluating cell-free DNA (cfDNA) screening test that can detect early cancer onset and predict tissue-of-origin.
**P13**	Patients with **prostate cancer** after radical treatment, evaluating genomic and epigenomic profile of tumor samples and cfDNA to predict clinical outcomes and effect on TME and tumor progression.
**P14**	Patients with **metastatic renal cell carcinoma**, following exposure to immunotherapy, evaluating changes in cfDNA methylation profiles at baseline and during treatment and correlate with disease response to immunotherapy.
**P20**	Patients with **recurrent or metastatic salivary gland tumors** treated with non-cytotoxic therapies, evaluating clinicopathological features, treatment outcomes and survival and to correlate them with the kinetics of ctDNA.
