# Supplementary material for: Immune profiling by bulk RNA-seq and multiplex immunofluorescence imaging across multiple cancer types
Source: Front Oncol. 2026 Aug 6;16:1876853. doi: 10.3389/fonc.2026.1876853 (PMC13489921; doi:10.3389/fonc.2026.1876853)
Supplement: Supplementary file 2 [file DataSheet2.pdf]

Supplementary table 2 – Mapping scheme detailing correspondence between specific xCell2 labels matched to each mIHC-defined population.

**Unified Cell Type**	**mIHC Marker**	**xCell2 Classification**
CD4 T cells	CD3+CD8-FoxP3-	CD4-positive, alpha-beta T cell
CD8 T cells	CD3+CD8+	CD4-positive, alpha-beta T cell
Treg	CD3+CD8-FoxP3+	regulatory T cell
CD68+ Macrophage	CD68+	Macrophage
B cells	CD20+	B cell
